# Affective forecasting in an orangutan: predicting the hedonic outcome of novel juice mixes

**DOI:** 10.1007/s10071-016-1015-0

**Published:** 2016-08-11

**Authors:** Gabriela-Alina Sauciuc, Tomas Persson, Rasmus Bååth, Katarzyna Bobrowicz, Mathias Osvath

**Affiliations:** Department of Philosophy, Cognitive Science, Lund University, Box 192, 221 00 Lund, Sweden

**Keywords:** Affective forecasting, Orangutans, Humans, Decision-making, Episodic memory, Animal planning

## Abstract

Affective forecasting is an ability that allows the prediction of the hedonic outcome of never-before experienced situations, by mentally recombining elements of prior experiences into possible scenarios, and pre-experiencing what these might feel like. It has been hypothesised that this ability is uniquely human. For example, given prior experience with the ingredients, but in the absence of direct experience with the mixture, only humans are said to be able to predict that lemonade tastes better with sugar than without it. Non-human animals, on the other hand, are claimed to be confined to predicting—exclusively and inflexibly—the outcome of previously experienced situations. Relying on gustatory stimuli, we devised a non-verbal method for assessing affective forecasting and tested comparatively one Sumatran orangutan and ten human participants. Administered as binary choices, the test required the participants to mentally construct novel juice blends from familiar ingredients and to make hedonic predictions concerning the ensuing mixes. The orangutan’s performance was within the range of that shown by the humans. Both species made consistent choices that reflected independently measured taste preferences for the stimuli. Statistical models fitted to the data confirmed the predictive accuracy of such a relationship. The orangutan, just like humans, thus seems to have been able to make hedonic predictions concerning never-before experienced events.

**Electronic supplementary material:**

The online version of this article (doi:10.1007/s10071-016-1015-0) contains supplementary material, which is available to authorized users.

## Introduction

Decisions and choices pervade our daily lives. In well-known situations prior experience guides us. But often, as we navigate through an ever-changing environment towards an inherently uncertain future, we find ourselves facing novel challenges. In such never-before encountered situations, our capacity for episodic constructive simulation comes to the rescue (as reviewed e.g. by Gilbert and Wilson 2007; Schacter et al. 2008; Schacter 2012). Equipped with this ability, humans can quickly conjure details from disparate memories and mentally construct never-before experienced situations. One crucial aspect of episodic simulation, which is captured by the notion of affective forecasting (henceforth AF), is that emotional responses are triggered as we mentally construct potential scenarios and envision their outcomes (e.g. Benoit et al. 2014). This allows us to pre-experience how these will make us feel (Gilbert and Wilson 2007, 2009). It turn, such imagination-driven emotions steer the choices we make, biasing us towards future events that feel good when we simulate them (Gilbert and Wilson 2007).

The adaptive significance of AF is obvious, as it saves the costs and risks of having to engage in actual behaviour to find out how novel situations might turn out (Gilbert and Wilson 2007; Schacter 2012). AF is hypothesised to be a human specialty; non-human animals, on the other hand, are said to be inflexibly constrained by prior experience. They can only learn—by trial-and-error—to predict the hedonic consequences of events they have experienced before (Gilbert and Wilson 2007). However, since AF research relies primarily on verbal reports (e.g. of predicted hedonic outcomes and experienced affective impact), the hypothesis that AF is unique to humans has not been tested directly. Yet, accumulating evidence from research on episodic memory and planning in other species, primarily great apes and corvids (as reviewed by Clayton 2014; Osvath and Martin-Ordas 2014; Scarf et al. 2014), suggests that other species too might possess the episodic abilities that are posited as a prerequisite for AF.

To test the hypothesis that other species than humans possess AF capabilities, we devised a non-verbal test of AF that allowed us to assess comparatively the performance of an orangutan and ten humans. Since previous research has demonstrated prospective cognition (e.g. planning) in orangutans (see Osvath and Martin-Ordas 2014, for a recent review), this species constitutes a good model for testing AF abilities in non-human subjects. As humans are the only species acknowledged to exhibit AF, they served as a control group for the non-human subject. The task relied on gustatory stimuli and was inspired by a series of food-related examples mentioned in the AF literature. For example, most humans are expected to predict that lemonade will taste better with sugar than without it (Wilson and Gilbert 2003), but to envision a liver popsicle as revolting (Gilbert and Wilson 2007). This process of mentally constructing novel food items by combining two familiar ones is found to selectively engage neural structures associated with episodic cognition (Barron et al. 2013). In our AF test, the participants were first familiarised with four distinctly coloured and distinctly flavoured liquids (henceforth ‘ingredients’) and then presented with binary choices between a familiar ingredient and a novel mix of two familiar ingredients (i.e. a never-before experienced combination). To verify that participants’ choices were guided by hedonic predictions, their choice-derived preferences in this task were compared with independent measures of taste preferences for the ingredients and mixes, collected after the main test. Crucial components of AF that are captured by the task are thus the ability to mentally construe novel gustatory events through flexible recombination of relevant memories and to predict their hedonic outcome.

## Methods

### General methods

The study consisted of four parts: (1) Familiarisation and ingredient preferences; (2) Affective forecasting test; (3) Control for colour biases in the orangutan’s performance in the AF test and (4) Independent post-experimental measures of taste preferences for ingredients and mixes (see Table 1 for an overview of the study).

**Table 1:** Overview of study phases

Phase	Brief description
Familiarisation and ingredient preferences	(a) Four ‘ingredient’ juices are selected from an initial battery of seven^a^. For this purpose, juices are paired two-by-two in binary choices. Blocked trials are administered with each pair until establishing those stimuli for which the subject shows a clear preference ranking. In this process, the orangutan is also familiarised with the ‘ingredient’ juices. Human participants received 30 familiarisation trials with the four preselected ingredients. Each of the six possible ingredient pairs are presented five times in blocked trials
	(b) To establish that participants clearly recognise the ‘ingredients’, they receive an additional number of 24 trials in which ingredient pairs occur in random order. Each ingredient pair occurs four times
Affective forecasting test	(a) ‘Transparent’ trials: participants are presented with binary choices between a familiar ingredient and a novel ‘mix’. The latter is obtained by combining, in front of the subjects, two familiar ingredients. By systematically mixing ingredients two-by-two, six novel mixes are obtained. By systematically pairing ingredients and mixes, 24 unique and novel choice contexts are derived. Participants have visual access to the ingredients and ensuing mix
	(b) ‘Concealed’ trials: participants are presented with binary choices between a familiar ingredient and a mix, but visual access to the liquids is obstructed before the mix is produced. Subjects can see which ingredients are involved and can see the experimenter pouring the contents of one bottle into another concealed bottle. They cannot see the ensuing mix and have to choose between two concealed bottles
Control for colour biases^a^	Colour–flavour associations for the ingredients are reversed. After an extinction phase, preferences are determined for ingredients presented in the reversed colours. These are compared to preferences for ingredients presented in the original colours
Post-experimental measures of taste preferences	An independent preference ranking for all ten liquids (ingredients and mixes) is established in a set-up in which these are presented in ‘disguise’ (in new colours) and mixes are presented pre-blended, having the appearance of novel ingredients. Blocked trials are administered for each unique pair of two liquids. Self-reported preference rankings are collected from the human participants

^a^Administered to the orangutan only

#### Participants

One male Sumatran orangutan (*Pongo abelii*) and ten humans (four females) took part in the study. The orangutan (Naong, born 1990) was 21 years old at the beginning of the study and was housed at Furuvik Zoo/Lund University Primate Research Station Furuvik in Sweden. His enclosure, comprising indoor quarters and outdoor island, was shared with a female of similar age. The female, who was newly arrived at the station and avoided unfamiliar humans, could not be involved in the study. Following the general policy of the research station, the orangutan engaged voluntarily in testing, by entering the experimental room, and was free to disengage at any time. The orangutan was tested across several days, roughly at the same time of the day, about 1–2 h after having had a meal.

The human participants (aged 20–35 years) were recruited and tested at Lund University, in Sweden. The call for participation mentioned the duration of the experiment and that it involved drinking small quantities of liquids, some of which were unpleasant to taste. After signing up, the participants were instructed not to consume any food or liquids prior to or during an experimental session. Participants were tested separately, in individual sessions. They were first acquainted with the set-up and presented with the instructions. The latter specified that the experiment consisted in making a choice between two small amounts of liquid and subsequently drinking (or at least tasting) the chosen liquid. The participants were also informed that they were free to verbalise throughout the experiment if they wished to do so. Finally, they were informed that they were free to quit the experiment at anytime and that their participation would be recompensed with cinema gift certificates. After having had the opportunity to ask questions concerning the experiment, the participants signed informed consent forms.

#### General procedure and materials

In each study phase, the participants were given a forced-choice task in which they could select between two liquids from a table, by using their hand, finger or a plastic straw. Liquid presentation was counterbalanced with respect to the position on the table. The liquids were presented in small plastic containers, in portions of 10 ml each. Given different testing conditions between the two sites (Lund University/Furuvik Zoo), we employed reusable bottles for the orangutan testing and disposable glasses for the human testing. The bottles and the glasses were comparable with respect to size and volume. In the orangutan set-up, the liquids were briefly presented outside the subject’s reach on a retractable table. The table was then pushed towards the subject so that he could make a choice, by extending a drinking straw (typically held between lips), towards one of the bottles; sometimes finger pointing was used. He was then allowed to drink the chosen liquid while the other bottle was removed from the table. The orangutan consumed the liquid with the help of the straw, through the cage bars. In the human set-up, participants were seated at a table, across the experimenter. The participants were explicitly instructed that as soon as they lifted a glass from the table, this would be recorded as a choice. Unlike the orangutan, they drank directly from the glasses. At both sites, water was freely available. The humans were provided with buckets for discarding non-ingested liquid.

Two experimenters were involved in conducting the orangutan testing—one experimenter prepared the stimuli and the other administered the task. During trial administration, the experimenter was silent and refrained from making head turns or gazing to the left or right, to avoid potential cueing. Only one experimenter conducted the human testing, as testing conditions at Lund University were less demanding.

The ingredient set included cherry juice, rhubarb juice, lemon juice, and diluted apple cider vinegar; this set was derived from an initial battery of seven liquids (see Online Resource 1 for more details on the selection procedure and results). In the orangutan testing, cherry and rhubarb juice were presented in their natural colour—red and pink, respectively. The colour of lemon juice and vinegar, which was similar for the two liquids, was altered to light green and dark green, respectively, by using food dyes. Since some (but not all) of the human participants were familiar with some of the colour–flavour associations (i.e. red-cherry and pink-rhubarb) used in the orangutan testing, a reversed colour scheme was employed in the human testing. Cherry juice was coloured in dark green, rhubarb juice in light green, vinegar in red, and lemon juice in pink. This ensured that all human participants were learning novel ingredient colour–flavour associations. The reversed colour scheme was also employed in part (3) of the study (Control for colour biases), which was administered to the orangutan only. The food dyes used for changing juice colours in the orangutan and human testing had no discernible taste that could have altered juice flavour.

### Familiarisation and ingredient preferences

To provide optimal materials for further testing, the aim of this initial phase was to ascertain that the participants were sufficiently familiarised with the ingredients.

#### Procedure and materials

The trials administered in this phase were instantiated by binary choice trials in which the four ingredients were paired with each other, thus forming six unique ingredient pairs. The human participants received 30 familiarisation trials in which each unique pair of ingredients occurred five times, in blocked trials. To ascertain that participants were sufficiently familiarised with the ingredients, they received an additional 24 trials (four trials/ingredient pair), in which ingredient pairs were presented in randomised order rather than in blocked trials, as previously.

For the orangutan, the preliminary phase of ingredient selection (see Online Resource 1 for more details) served also to familiarise the subject with the experimental ingredient set. After ingredient selection/familiarisation, just like the human participants, the orangutan received 24 randomised trials with the six ingredient pairs. He received an additional 26 such randomised trials in the middle of the AF test, as well as before the colour control.

#### Results

To ensure that participants were sufficiently familiarised with the ingredients, choice-derived preferences in the blocked trials were compared with choice-derived preferences in the randomised trials. Preference scores were computed as percentages representing the number of times an ingredient was chosen across all occasions in which it was encountered. Individual ingredient preferences did not differ significantly across the two set-ups (all *Ps* > 0.05, range 0.11–1, Fisher’s exact test). This suggested that all participants had been sufficiently familiarised with the ingredients and had formed stable preferences for them.

### Affective forecasting test

In order to probe their AF ability, participants were presented with a task whereby novel choice situations were systematically created by pairing a familiar ingredient with a novel mix, which was obtained by combining two familiar ingredients. By administering this task, we sought to examine how participants responded when confronted with novel juice mixes. More specifically, the aims were (1) to obtain a preference ranking for ingredients and mixes; (2) to assess whether subjects were consistent in their choices; and (3) to rule out the presence of certain biases (novelty, volume). A central prediction of the hypothesis that only humans possess AF is that a non-human animal will exhibit trial-and-error performance upon its first encounters with never-before experienced situations. In the context of our task, this can be measured by assessing whether the orangutan subject exhibits random as opposed to consistent choices across the first and second encounters with each novel ingredient-mix pair. In this assessment, random choices would be indicative of trial-and-error performance. Evidence of choice constancy, on the other hand, would suggest an ability to make principled choices even when confronted with never-before experienced stimuli and contexts. Note, however, that choice consistency is an insufficient criterion for establishing the presence of an ability to make *hedonic* predictions concerning novel experiences, as non-hedonic criteria might also underlie consistent choices. For example, the orangutan could have chosen based on the novelty of the mixes or showed a bias towards avoiding (or preferentially choosing) the mix. Moreover, given different portion size for the two liquids presented in each AF test trial (as detailed below), the subject could have been biased towards choosing the larger portion.

#### Procedure

As in the *Familiarisation and ingredient preferences,* the participants were administered a binary forced-choice task. By systematically pairing familiar ingredients with novel mixes, 24 novel and unique ingredient-mix pairs were obtained. Each subject received a total of 96 trials in which the 24 ingredient-mix pairs were presented in randomised order. Each unique ingredient-mix pair occurred four times, but typically only once every 24 trials. The task was administered in two conditions: *transparent* (trials 1–48) and *concealed* (trials 49–96).

**Fig. 1:**
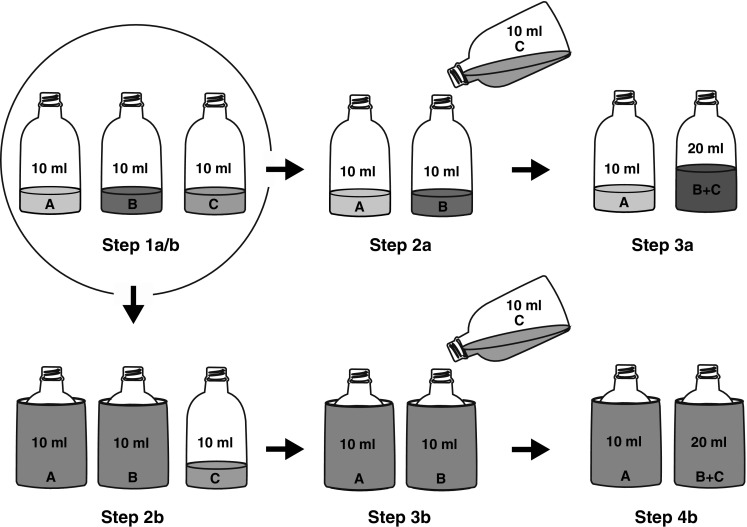
Procedure employed in the AF test. The *top series* illustrates a ‘transparent’ trial. *Step 1a/b*: the subject is presented with three ingredients. *Step 2a*: two of the ingredients are mixed in front of the subject to obtain a never-before experienced mix. *Step 3a*: the subject makes a choice between a familiar ingredient and a novel mix. The *bottom series* illustrates a ‘concealed’ trial. *Step 1a/b*: the subject is presented with three ingredients. *Step 2b*: the contents of two bottles are concealed. *Step 3b*: the content of the third bottle is poured into one of the concealed bottles. *Step 4b*: the subject is to make a choice between two concealed bottles, one containing a familiar ingredient and the other a novel mix

In the *transparent* condition, the participants had constant visual access to the liquids contained in the bottles. In each of these trials, three bottles, each containing 10 ml of an ingredient, were placed on the table (Fig. 1, Step 1a/b). The content of one bottle was then poured into an adjacent bottle, so that two ingredients were mixed in front of the participants resulting into a novel drink (Fig. 1, Step 2a). The empty bottle was removed from the table and the participants had to choose between 10 ml of a familiar ingredient and 20 ml of a novel mix (Fig. 1, Step 3a).

In the *concealed* condition, to increase the demands for mental representation in the absence of tangible information, visual access to the stimuli was obstructed before the mix was produced by the experimenter. More specifically, the participants were allowed quick visual access (typically 5–10 s) to the three bottles containing ingredients (Fig. 1, Step 1a/b), after which the contents of the bottles were concealed (Fig. 1, Step 2b). The participants did thus not witness the actual mixing of the ingredients nor did they witness the ensuing mix; they could, however, see that the content of one bottle was poured into another, concealed, one (Fig. 1, Step 3b). After at least 8 s had elapsed from the last visual access to the content of the bottles, the participants were given an opportunity to choose between two concealed bottles (Fig. 1, Step 4b). This set-up prevents learnt colour–taste associations for the mixes from driving choices and constrains the participants to form and keep a representation of the stimuli active in working memory, i.e. beyond the two-second window of sensory short-term memory (as reviewed by Carruthers 2013).

Before engaging in the task, the orangutan received a total of 27 training trials. In 15 of these, it was ascertained that he was able to understand that liquid volume remained equal when poured into a concealed container. These 15 trials were binary choices between familiar ingredients. The remaining 12 trials were aimed at ascertaining that the juice-mixing event—given its salience—would not engender novelty biases for the subject. Non-experimental juices were used in these trials, including three liquids discarded during the ingredient selection phase (blueberry juice, strawberry juice, salt water), and a fourth added one (artichoke). The first six of these 12 trials were binary choices between ingredients (similar to the randomised trials in *Familiarisation and ingredient preferences*), to determine that the subject recognised them. The last six trials introduced the novel procedure in which binary choices paired a familiar ingredient with a novel mix. The subject did not show a bias for ingredients or mixes, but selected them an equal amount of times.

#### Results

**Fig. 2:**
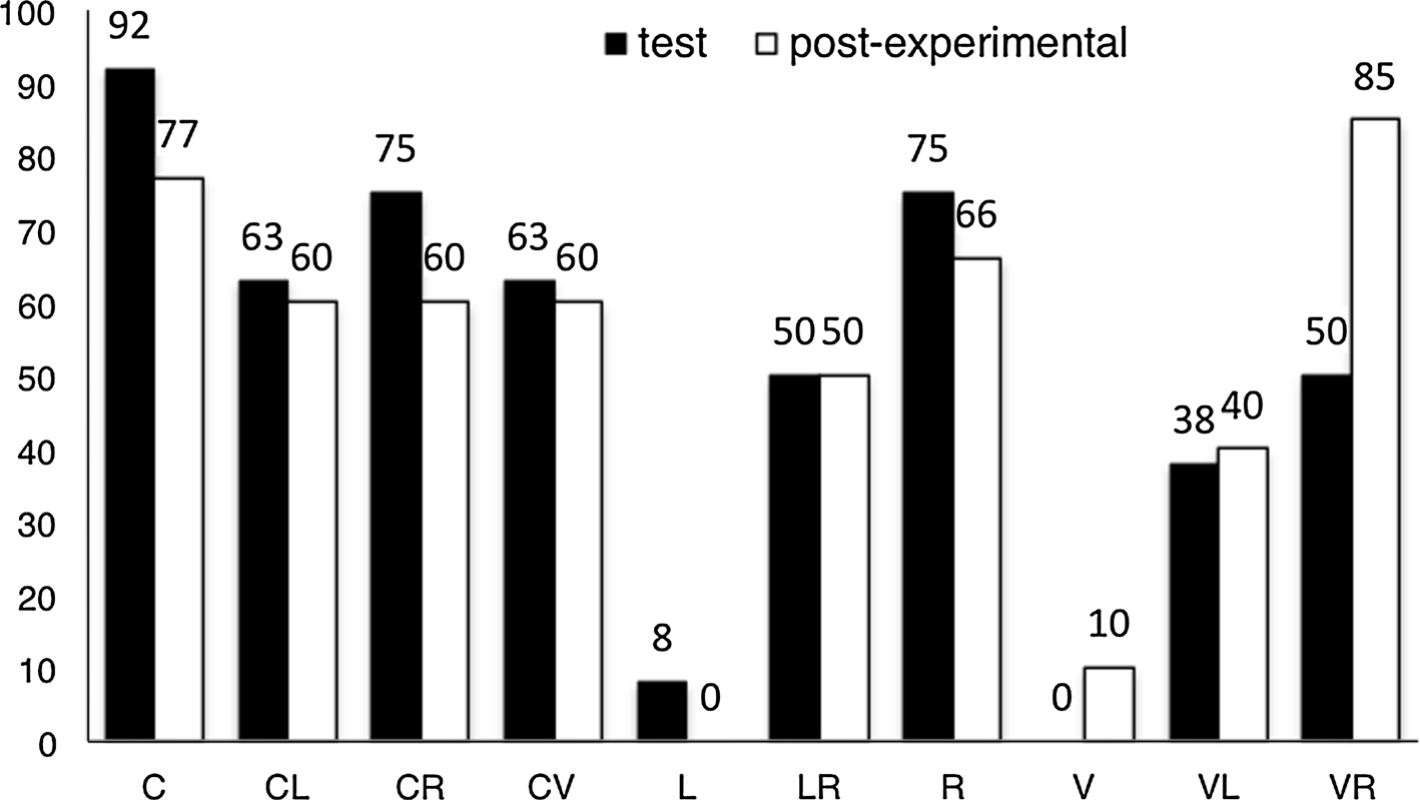
Orangutan’s preferences based on his choices in the first and second encounters with each novel ingredient-mix pair compared to post-experimental preferences. Preferences are presented as proportion of times each item was chosen across all occasions in which it was encountered. *C*: cherry juice, *CL*: cherry and lemon mix, *CR*: cherry and rhubarb mix, *CV*: cherry and vinegar mix, *L*: lemon juice, *LR*: lemon and rhubarb mix, *R*: rhubarb juice, *VL*: vinegar and lemon mix, *VR*: vinegar and rhubarb mix, *V*: vinegar

**Fig. 3:**
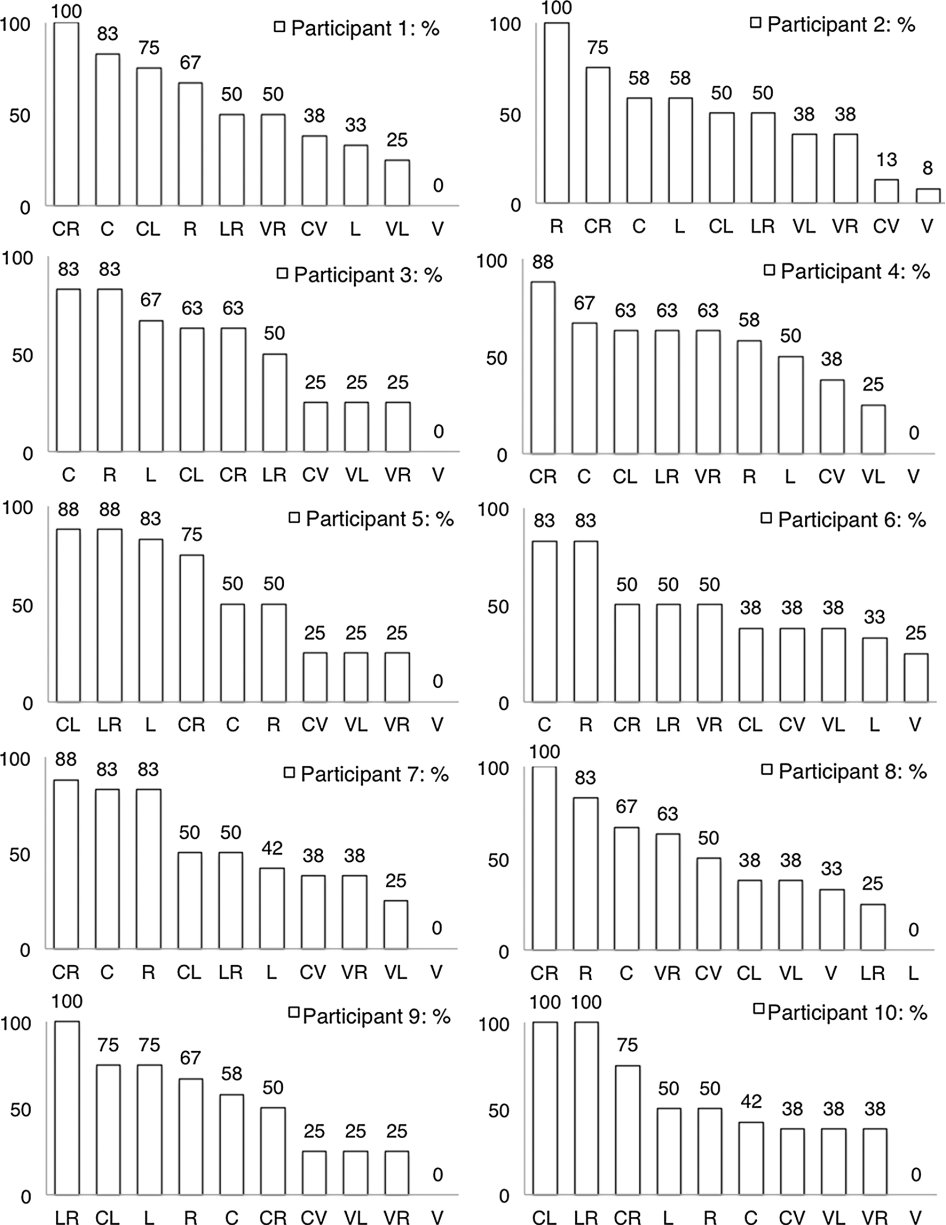
Preferences of human participants based on choices made in the first and second encounters with each novel ingredient-mix pair. Preferences are presented as proportion of times each item was chosen across all occasions in which it was encountered. *C*: cherry juice, *CL*: cherry and lemon mix, *CR*: cherry and rhubarb mix, *CV*: cherry and vinegar mix, *L*: lemon juice, *LR*: lemon and rhubarb mix, *R*: rhubarb juice, *V*: vinegar and lemon mix, *VR*: vinegar and rhubarb mix, *V*: vinegar

*Test-derived individual preferences for ingredients and mixes* In the experimental set of 24 novel ingredient-mix pairs, ingredients and mixes occurred an unequal number of times, with each of the four ingredients occurring more often than the six ensuing mixes. For this reason, individual preference scores for each of the ten liquids were computed as percentages representing the total number of times a given liquid was chosen in the total number of occasions in which it was encountered in the first and second trials for each unique ingredient-mix pair. Individual preference scores and a preference ranking are presented in Fig. 2 for the orangutan and in Fig. 3 for the ten human participants.

*Choice consistency* Across the first and second encounters with each novel ingredient-mix pair, the orangutan chose identically in 88 % cases (21 of 24 possible pairs), which is significantly different from chance (*P* < 0.001, binomial test). Choice consistency for the human participants ranged from 71 to 92 % (17–22 constant choices of 24 possible), being significantly different from chance for eight individuals (*Ps* ≤ 0.02, binomial test) and closely approaching significance for the remaining two (*P* = 0.06, see Table 2 for more details). To determine if there were cross-species differences with respect to choice consistency, the orangutan’s performance was compared, separately, with the performance of each human participant. We found the orangutan’s performance to be similar to that of humans’ (all *P*s ≥ 0.29, Fisher’s exact test).

**Table 2:** Level of choice consistency: (1) in the first two encounters with each novel ingredient-mix pair; (2) across the transparent and concealed conditions; and (3) in the concealed condition

Individual	First two encounters		Across conditions		Concealed condition	
	% Consistent	Comparison to chance^a^	% Consistent	Comparison to chance^a^	% Consistent	Comparison to chance^a^
Orangutan	88	<0.01	82	<0.01	90	<0.01
P1	79	<0.01	100	<0.01	100	<0.01
P2	79	<0.01	91	<0.01	91	<0.01
P3	75	0.02	83	<0.01	96	<0.01
P4	71	0.06	83	<0.01	79	<0.01
P5	92	<0.01	96	<0.01	96	<0.01
P6	71	0.06	63	0.31	79	<0.01
P7	88	<0.01	92	<0.01	100	<0.01
P8	88	<0.01	96	<0.01	96	<0.01
P9	92	<0.01	96	<0.01	88	<0.01
P10	79	<0.01	96	<0.01	83	<0.01

^a^Binomial test

Choice consistency was further assessed across *transparent* and *concealed* trials, as well as within the *concealed* trials. Across first *concealed* and last *transparent* trials for each unique ingredient-mix pair, the orangutan’s level of consistency was 82 % (*P* < 0.01, binomial test). All human participants but one showed similar high levels of consistency, ranging between 83 and 100 % (all *P*s < 0.01, see Table 2 for more details). Within the *concealed* condition, the orangutan’s level of consistency was 90 % (*P* < 0.001, binomial test); level of consistency for the ten human participants ranged from 79 to 100 % (all *Ps* < 0.01, see Table 2 for more details).

*Control for volume and novelty biases* To rule out the possibility that such biases affected the orangutan’s choices in the AF test, we verified if the orangutan showed a preference for ingredients (or conversely mixes) in these trials. In the first and second trials for each unique ingredient-mix pair, the orangutan chose ingredients in 21 cases and chose mixes in the remaining 27 (*P* = 0.48, binomial test). Likewise, across all 96 trials that were administered in the AF test, the ratio of mix versus ingredient choices was 55–41, indicating that there was no significant preference for the novel versus familiar type of stimulus nor for the larger volume of liquid (*P* = 0.18, binomial test). The human participants chose on average 23.5 ingredients (range 17–28) and 24.5 mixes (range 30–31). Separate comparisons between the orangutan and each human participant showed no significant differences concerning choice distribution between ingredients and mixes in the first two encounters with each novel ingredient-mix pair (all *Ps* > 0.05, range 0.22–1, Fisher’s exact test).

### Control for colour biases in the orangutan’s performance in the AF test

Since ingredient selection led to an ingredient set that included exclusively sweet liquids in the red colour spectrum and sour liquids in the green spectrum, it was important to control for the possibility that colour biases affected the subject’s choices. According to the red–green axis hypothesis, primate trichromacy is an adaptation to a feeding ecology that involves the detection of potential food sources food (ripe fruits, young leaves) from the rarely consumed green mature foliage. In line with this hypothesis, human experiments that employ small stimulus sets show that green colouring increases the perceived sourness of stimuli, while red colouring increases their perceived sweetness; such biases, however, are not present when large stimulus sets are employed (e.g. Spence et al. 2010, for a review). A study with Borneo orangutans (*Pongo pygmaeus*) suggests that colour biases might affect non-human apes as well, since one juvenile individual was found to consume more of the same food when this was coloured in red (Barbiers 1985).

#### Subject

The colour control was administered to the orangutan subject, which, as a representative of a non-human species, is the focal subject of the study. The human participants did not receive a similar control task, since the presence of AF in humans is not contested. Instead, the human participants served as a control group for assessing whether the orangutan’s performance in the key AF test was comparable to that of humans’.

#### Materials and procedure

To control for the possibility that the subject preferentially chose red juices (and their combinations) over green ones on the basis of their colour rather than their taste, ingredient colours were reversed after the completion of the AF test. Using food dyes, cherry juice was coloured in dark green, rhubarb juice in light green, vinegar in red and lemon juice in pink. Following a brief phase in which original colour–flavour associations were extinguished (see Online Resource 2 for more details), the subject received 36 trials in order to establish choice-derived preferences for the ingredients presented in reversed colours. These preferences were compared with ingredient preferences derived from choices in the preliminary phase, when ingredients were presented in their ‘original’ colour. The procedure was similar to the one in the last phase of *Familiarisation and ingredient preferences*. Each of the six possible ingredient pairs were presented in randomised order and occurred six times.

#### Results

A comparison of choices of the ingredients presented in their original colour with choices of ingredients presented in reversed colours revealed no significant differences across the two stimulus variations (*P* = 0.59 Fisher’s exact test). Indeed, in the ‘original’ ingredient preference trials the orangutan chose sweet drinks in 76 % of the trials, while in the trials with reversed colours he chose sweet drinks in 83 % of the trials. The results indicate that subject’s choices in the AF test were not affected by colour biases in line with the red–green axis hypothesis.

Summing up the results thus far, we established that the orangutan performed non-randomly when presented with novel mixes and novel choice contexts and that his performance was within the range of that shown by the humans. We further ruled out the possibility that certain non-hedonic criteria—including novelty, volume or colour—underlie his consistent choices in the first encounters with novel mixes and novel choice contexts.

### Independent post-experimental measures of taste preferences for ingredients and mixes

The aim of this final part of the study was to determine if participants’ choices when presented with novel mixes (in the AF test) were motivated by hedonic forecasts, i.e. by how the mixes were predicted to taste. For this purpose, separate measures of taste preferences were obtained from the participants, in the absence of additional task demands, such as ingredient mixing. These were then compared to choice-derived preferences in the first and second encounters with the novel ingredient-mix pairs in the AF test. Finding a relationship between the two preference measures would indicate that participants’ performance in the AF test was supported by a mental process that maximised the likelihood of selecting the most pleasant outcome.

#### Procedure and materials

An independent preference ranking for the four ingredients and the six ensuing mixes was obtained from the human participants by means of self-report. More specifically, they were asked to rank the ten liquids from most to least preferred. This also allowed us to corroborate taste preferences based on behavioural responses (i.e. participants’ choices in the AF test), with self-reported preferences after task completion, i.e. after novel juices have been experienced several times. This procedure parallels a commonly employed approach in AF research, whereby self-reports of predicted hedonic outcomes for certain events are compared with self-reports of experienced hedonic impact of those events.

The orangutan was presented with a new set of binary choices in which ingredients and mixes were contrasted pairwise in blocked trials. Crucially, in the post-experimental preference trials, the ten juices were presented in ‘disguise’. The ingredients were reversed to their original colour, and the mixes were randomly assigned new colours, such as yellow (lemon–vinegar), orange (cherry–rhubarb), light blue (rhubarb–lemon), dark blue (cherry–lemon), brown (cherry–vinegar), and milky green (rhubarb–vinegar). Furthermore, the mixes were presented pre-blended, thus taking the appearance of novel ingredients. Liquids in a pair were now presented in equal portions of 10 ml each. Prior to administering the first trial of each block, the subject was allowed to sample each liquid in the respective pair. There were typically five trials in each block, so that each unique pair of liquids occurred typically five times. A preference ranking was then derived based on scores representing the percentage of times a stimulus was chosen across all the pairs in which it occurred.

#### Results

To verify that hedonic predictions guided participants’ choices in the AF test, choice-derived preferences in the first two encounters with each novel ingredient-mix pair were compared with post-experimental preferences. The latter are summarised in Fig. 2 for the orangutan and Table 3 for the human participants. As this comparison relied on a small set of categorical data and tied ranks were expected, Kendall’s tau-b correlation coefficients were computed to establish whether the two preference measures were related (e.g. Agresti 2010). We found the orangutan’s preferences in the first two encounters with each ingredient-mix pair in the AF test to correlate highly and significantly with post-experimental preferences (*τ*_*b*_ = 0.67, *P* = 0.01, *N* = 10); a similar result was found for choices in the concealed trials (*τ*_*b*_ = 0.68, *P* = 0.008, *N* = 10). Collapsing ‘transparent’ and ‘concealed’ trials (i.e. all 96 test trials), we found task choices to correlate highly and significantly with post-experimental choices (*τ*_*b*_ = 0.71, *P* = 0.006, *N* = 10).

**Table 3:** Post-experimental preference measures: human participants

Item	P1	P2	P3	P4	P5	P6	P7	P8	P9	P10
C	1	3	5	1	5	1	1	3	6	5
CL	4	4	4	4	2	4	4	8	4	6
CR	3	2	3	3	4	2	2	2	3	4
CV	10	8	8	7	8	6	8	5	8	7
L	6	6	6	6	3	7	6	9	1	3
LR	5	5	1	5	1	5	5	7	2	2
R	2	1	2	2	6	3	3	1	5	1
VL	7	9	9	8	7	9	7	10	9	9
VR	8	7	7	10	9	8	9	4	7	8
V	9	10	10	9	10	10	10	6	10	10

*C*: cherry juice, *CL*: cherry and lemon mix, *CR*: cherry and rhubarb mix, *CV*: cherry and vinegar mix, *L*: lemon juice, *LR*: lemon and rhubarb mix, *R*: rhubarb juice, *VL*: vinegar and lemon mix, *VR*: vinegar and rhubarb mix, *V*: vinegar

1, most preferred; 10, least preferred

Similarly, for the human participants, test-derived preferences in the first two encounters with each novel ingredient-mix pair correlated highly and significantly with self-reported preferences, with correlation coefficients ranging from *τ*_*b*_ = 0.52 (*P* = 0.04, *N* = 10) to *τ*_*b*_ = 0.94 (*P* < 0.001, *N* = 10, see Table 4 for more details). Likewise, choice-derived preferences in the concealed trials correlated significantly with self-reported preferences: *τ*_*b*_ ranged from 0.54 (*P* = 0.04, *N* = 10) to 0.89 (*P* < 0.001, *N* = 10).

**Table 4:** Correlation between test-derived preferences in the first two encounters with each novel ingredient-mix pair and post-experimental preference measures

Naong	P1	P2	P3	P4	P5	P6	P7	P8	P9	P10
0.67	0.63	0.83	0.52	0.55	0.94	0.63	0.83	0.72	0.77	0.61

All correlations are significant at *P* < 0.05

For the orangutan data, two Bradley–Terry models (Bradley and Terry 1952) were further implemented in order to estimate the predictive accuracy of the hypothesis that choices in the first two encounters with the novel ingredient-mix pairs were driven by hedonic predictions. This statistical approach is often applied to pairwise comparison data for the purposes of individual preference modelling. The assumptions of a Bradley–Terry model are that the data consist of paired choices and that, for each choice, the probability of choosing one item over the other depends on the subjective value of that item compared to the other item. This value is an unknown parameter that is estimated using the data. The two Bradley–Terry models were estimated using the bbmle package for R (Bolker 2008) where the difference between the models consists in how the subjective values are assigned. In model A, subjective values for each ingredient or mix were estimated based on the assumptions that subjective values did not change across relevant trials, i.e. first and second trials with each ingredient-mix pair in the test and post-experimental trials. Model B extended model A by estimating separate values for the first two times a specific novel pair was encountered and for the rest of the trials. Model A is consistent with the assumption that choices in the first two encounters with each ingredient-mix pair were guided by predictions concerning taste preferences. Model B, on the other hand, would better fit that data if the test trials examined were not driven by predicted taste preferences, thus differing from post-experimental choices. Three measures were used to compare the two models and all pointed to model A as being a better fit than model B, thus favouring the model assuming that hedonic predictions explain choices in the examined trials. A comparison of the two models using the Akaike information criterion (Akaike 1981) favoured model A (AIC = 143) over model B (AIC = 152), as did a comparison using the Bayesian information criterion (Schwarz 1978) with model A having a BIC of 173 and model B having a BIC of 202. Further, a likelihood ratio test showed no statistically significant improvement of using model B over model A [*χ* ^2^(6) = 2.64, *P* = 0.85].

## Discussion

Affective forecasting enables individuals to predict the hedonic outcome of novel situations by mentally recombining elements of prior experiences into possible scenarios, and pre-experiencing what these might feel like. This ability is hypothesised to have evolved in the hominin lineage and hence to be absent in any other extant animals than humans.

In this study, we presented an orangutan and ten humans with a novel, non-verbal, AF test that relied on gustatory stimuli. Four familiar ingredients were combined to form six never-before experienced mixes that were presented in 24 unique comparison contexts. By the nature of the stimuli, and by the structure and demands of the task, if the participants were to perform efficiently, they had to mentally integrate relevant memories to generate novel liquid blends and predict their hedonic consequences. Mental taste blending has been repeatedly given as a prime example of AF (e.g. Wilson and Gilbert 2003; Gilbert and Wilson 2007). Moreover, in humans, who are the only species known to use AF, this process is shown to engage episodic simulation, as well as mechanisms of abstract valuation, i.e. which allow the evaluation of mentally constructed outcomes (Barron et al. 2013).

We found that the orangutan made consistent choices when confronted with never-before experienced situations, rather than responding randomly (i.e. by trial-and-error). Moreover, his consistent choices were predicted by independently collected taste preferences for ingredients and mixes. In turn, this indicates that in the AF test, in the first encounters with novel ingredient-mix pairs, his choices were guided by predictions concerning the hedonic outcome of the mixes. Overall, the orangutan’s performance was comparable to that of the human participants. Importantly, and further suggesting that our behavioural task indeed taps into AF, task-derived taste preferences were corroborated by self-reported preferences collected from the human participants.

We considered—and ruled out—a number of alternative and arguably simpler strategies that could have accounted for the orangutan’s performance. We showed that choice consistency was not due to a familiarity or novelty bias as, in the AF test, the orangutan did not chose the familiar ingredient significantly more often than the novel mix nor vice versa. Likewise, he did not show a preference for the larger volume. We further showed that colour biases in line with a red–green axis hypothesis were unlikely to influence the orangutan’s choices in the AF test. Moreover, we excluded the possibility that these choices were driven by the ensuing colour of the mix, as the procedure was adjusted to cut visual access to the ensuing mixes and, in fact, to both choice items. This set-up did not affect the orangutan’s performance: his choices in *concealed* trials were consistent with those in *transparent* trials, and were predicted by independently collected post-experimental preferences. Yet another potential explanation to consider is whether the orangutan’s performance could have been accounted for by an ‘ingredient-tracking’ strategy, whereby he would always select cherry (the most preferred ingredient) or always avoid lemon (the least preferred ingredient), regardless of the mix in which it occurred, or irrespective of the other choice item. Such a strategy predicts, for example, that a less-preferred item (rhubarb) would never be selected when cherry is brought to the table. Yet, this was not the case as the subject did select rhubarb over a mix of cherry–lemon or cherry–vinegar, but not over a mix of cherry–rhubarb. Conversely, there was no evidence of consistent avoidance of lemon, as the orangutan chose mixes involving this ingredient in a relative manner, i.e. depending on the other choice item. In any case, such an ingredient-tracking strategy would fail to explain the significant correlation between test-derived and post-experimental preferences. In the post-experimental trials, the subject no longer had the possibility to track ingredients in the mixes, as these were presented pre-blended and ‘disguised’ by new colours.

To test the presence of hedonic forecasts, data analysis focused on first-trial performance for each of the 24 unique ingredient-mix pairs. Participants’ first direct exposure to the mixes occurred only *after* the completion of a decision-making process that required mental construction and hedonic prediction. Moreover, novel mixes were successively presented in novel choice contexts, and each of these required flexible access to memories, as well as the construction of inferred outcomes and prospective values. In fact, the combinatorial demands of the procedure, involving six novel items occurring randomly in 24 different choice contexts, and always requiring new value computations, should be sufficiently high to preclude trial-and-error learning of the novel combinations and their relative values.

Taken together, the results are consistent with a view that concedes the orangutan AF capabilities. Just like the human participants in our experiment or the hypothetical human portrayed in examples from the AF literature (e.g. Wilson and Gilbert 2003), the orangutan was indeed able to predict that lemon juice tastes better when sweetened. Given the similar performance of the two species, the evidence that humans engage episodic mechanisms when performing a task similar to ours, and the similarity of relevant neural architecture between closely related species, the most parsimonious explanation for the results presented here is that the orangutan evidenced AF. In turn, this challenges the hypothesis that AF is an ability restricted to humans and suggests ancient evolutionarily roots for this crucial human ability. Since only one non-human subject was tested in this study, we acknowledge, however, the limitations of the conclusions that can be drawn from this study. We hope that our results will attract more efforts towards a diversification of both focus and approaches, thus allowing for a better understanding of varieties of prospection, their specificities and underlying mechanisms, in humans as well as in other species.

## Electronic supplementary material

Below is the link to the electronic supplementary material.

Supplementary material 1 Online resource 1: Ingredient selection and familiarisation - detailed procedure and results (PDF 102 kb)

Supplementary material 2 Online resource 2: Extinction phase preceding control for colour biases - procedure and results (PDF 95 kb)
