# Supplementary material for: Affective forecasting in an orangutan: predicting the hedonic outcome of novel juice mixes
Source: Anim Cogn. 2016 Aug 11;19(6):1081–92. doi: 10.1007/s10071-016-1015-0 (PMC5054047; doi:10.1007/s10071-016-1015-0)
Supplement: Supplementary file 1 — Online resource 1: Ingredient selection and familiarisation - detailed procedure and results (PDF 102 kb) [file 10071_2016_1015_MOESM1_ESM.pdf]

Affective forecasting in an orangutan - predicting the hedonic outcome of novel juice mixes

Journal: *Animal Cognition*

Authors: Sauciuc G, Persson T, Bååth R, Bobrowicz K, Osvath M

Corresponding author: Gabriela-Alina Sauciuc

Affiliation: Lund University, Department of Philosophy, Cognitive Science

E-mail address: Gabriela-Alina.Sauciuc@lucs.lu.se

Online Resource 1

Ingredient selection and familiarisation - detailed procedure and results

Ingredient liquids were derived from an initial battery of seven distinctly flavoured and distinctly coloured liquids. These instantiated both pleasant (sweet) and purportedly less pleasant (salt, sour) tastes, including commercially available fruit juices (cherry, strawberry, blueberry and rhubarb), as well as sodium chloride solution (salt water), diluted lemon juice and diluted apple cider vinegar. The sodium chloride solution was presented at 2% concentration; the sour drinks (lemon juice and vinegar) were diluted (1:9) with water. Liquids that were similar in colour were altered using food dyes. Strawberry juice was coloured in yellow, blueberry juice was coloured in dark blue, salt water in light blue, vinegar in dark green, lemon juice in light green. Cherry juice and rhubarb retained their natural red, respectively pink, colours.

The procedure consisted in contrasting the 7 liquids pairwise, in binary choices. The orangutan was already familiar with choice tasks but he was naïve to the stimuli and all other

procedural details of the experiment. Liquids were first paired within the same purported hedonic spectrum, i.e. sweet liquids were paired with other sweet liquids and unpleasant liquids (salt water, vinegar and lemon juice) were paired with one another. After administering 49 trials using this procedure, we could identify the two hedonic extremes. These were cherry juice (the most preferred of the seven liquids) and lemon juice (the least preferred). Additionally, cherry was unambiguously preferred to rhubarb. More specifically, cherry juice was 100% preferred to rhubarb juice (5 trials), and 70% preferred to strawberry juice (10 trials); blueberry juice was 73% preferred to strawberry juice (11 trials). Conversely, lemon juice was not preferred to salt water (7 trials) or apple cider vinegar (4 trials). The orangutan did not show a clear preference when the paired juices were cherry - blueberry (6 trials), or strawberry - rhubarb (6 trials). Subsequently, rhubarb juice was contrasted separately with lemon (6 trials), vinegar (5 trials) and salt water (5 trials). After noticing that salt water was 100% preferred to rhubarb juice, apple cider vinegar was retained as an experimental stimulus and salt water was, for at least two reasons, discarded. Firstly, this preference might have reflected a momentary craving and thus, later in the experiment, give rise to unstable choices, compromising the experiment. Secondly, and taking into account ethical concerns, if these preliminary trials reflected a stable preference rather than a momentary craving, there was a risk for salt overconsumption by the subject. This ingredient selection phase was concluded by 5 additional trials in which cherry was contrasted with lemon. Throughout the phase, stimulus presentation (left-right) was counterbalanced. Besides allowing us to select an ingredient set, this preliminary phase served also to familiarize the orangutan with the liquids.
