# Supplementary material for: Affective forecasting in an orangutan: predicting the hedonic outcome of novel juice mixes
Source: Anim Cogn. 2016 Aug 11;19(6):1081–92. doi: 10.1007/s10071-016-1015-0 (PMC5054047; doi:10.1007/s10071-016-1015-0)
Supplement: Supplementary file 2 — Online resource 2: Extinction phase preceding control for colour biases - procedure and results (PDF 95 kb) [file 10071_2016_1015_MOESM2_ESM.pdf]

Affective forecasting in an orangutan - predicting the hedonic outcome of novel juice mixes

Journal: *Animal Cognition*

Authors: Sauciuc G, Persson T, Bååth R, Bobrowicz K, Osvath M

Corresponding author: Gabriela-Alina Sauciuc

Affiliation: Lund University, Department of Philosophy, Cognitive Science

E-mail address: Gabriela-Alina.Sauciuc@lucs.lu.se

Online Resource 2

Extinction phase preceding control for colour biases: procedure and results

To extinguish previously acquired colour-flavour associations, the four ingredients - now presented in 'reversed' colours - were contrasted pairwise (in six unique ingredient pairs) in block trials. A total of 101 trials were administered for this purpose. The number of trials administered with each of the six ingredient pairs varied, depending on when extinction was achieved. The success criterion was set at 80% consistent choices in 5 consecutive trials.

We tracked three different stages during the extinction process, which were labelled as 'learning', 'inferential' and 'consolidation'. The 'learning' stage includes those trials in which the subject was acquiring the 'reversed' colour-flavour associations, including the 5 consecutive trials in which the subject passed the success criterion. There were 62 such learning trials, and these involved three of the six possible ingredient pairs (cherry-vinegar, rhubarb-lemon, and rhubarb-vinegar). Of these, 14 trials were administered with the cherry-vinegar pair, 30 trials with the rhubarb-lemon pair, and 18 trials with the vinegar-rhubarb pair. The 'inferential' stage

comprised 18 trials in which the orangutan was presented with the remaining three ingredient pairs (cherry-lemon, cherry-rhubarb, and lemon-vinegar). In these trials, the subject spontaneously transferred the colour-flavour associations acquired with the first three pairs (cherry-vinegar, rhubarb-lemon and rhubarb-vinegar) to the remaining ingredient pairs, i.e. cherry-lemon, cherry-rhubarb and vinegar-lemon. The ‘consolidation’ stage comprised 21 trials in which the newly acquired colour-flavour associations was re-assessed and confirmed.
